# Supplementary material for: FAM46C/TENT5C functions as a tumor suppressor through inhibition of Plk4 activity
Source: Commun Biol. 2020 Aug 17;3:448. doi: 10.1038/s42003-020-01161-3 (PMC7431843; doi:10.1038/s42003-020-01161-3)
Supplement: Supplementary file 1 — Supplementary Information [file 42003_2020_1161_MOESM1_ESM.pdf]

FAM46C/TENT5C functions as a tumour suppressor through inhibition of Plk4 activity.

SI APPENDIX

Supplementary Figures

Supplementary Figure 1

A

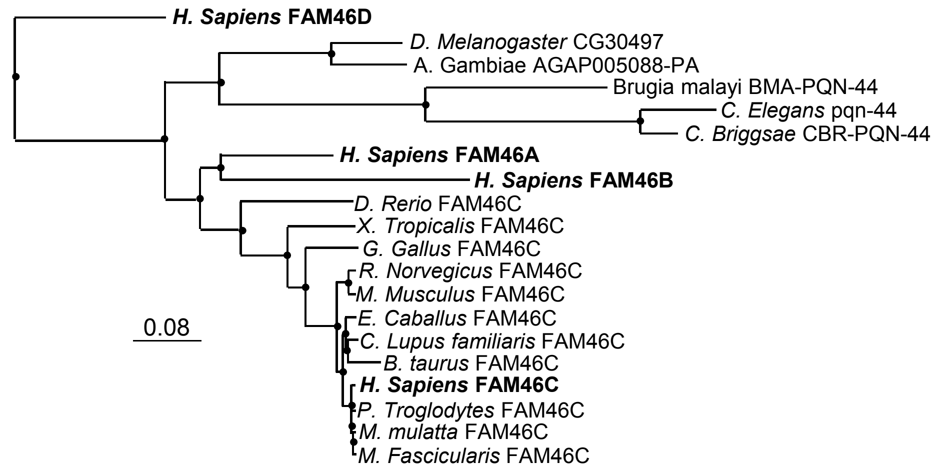

B

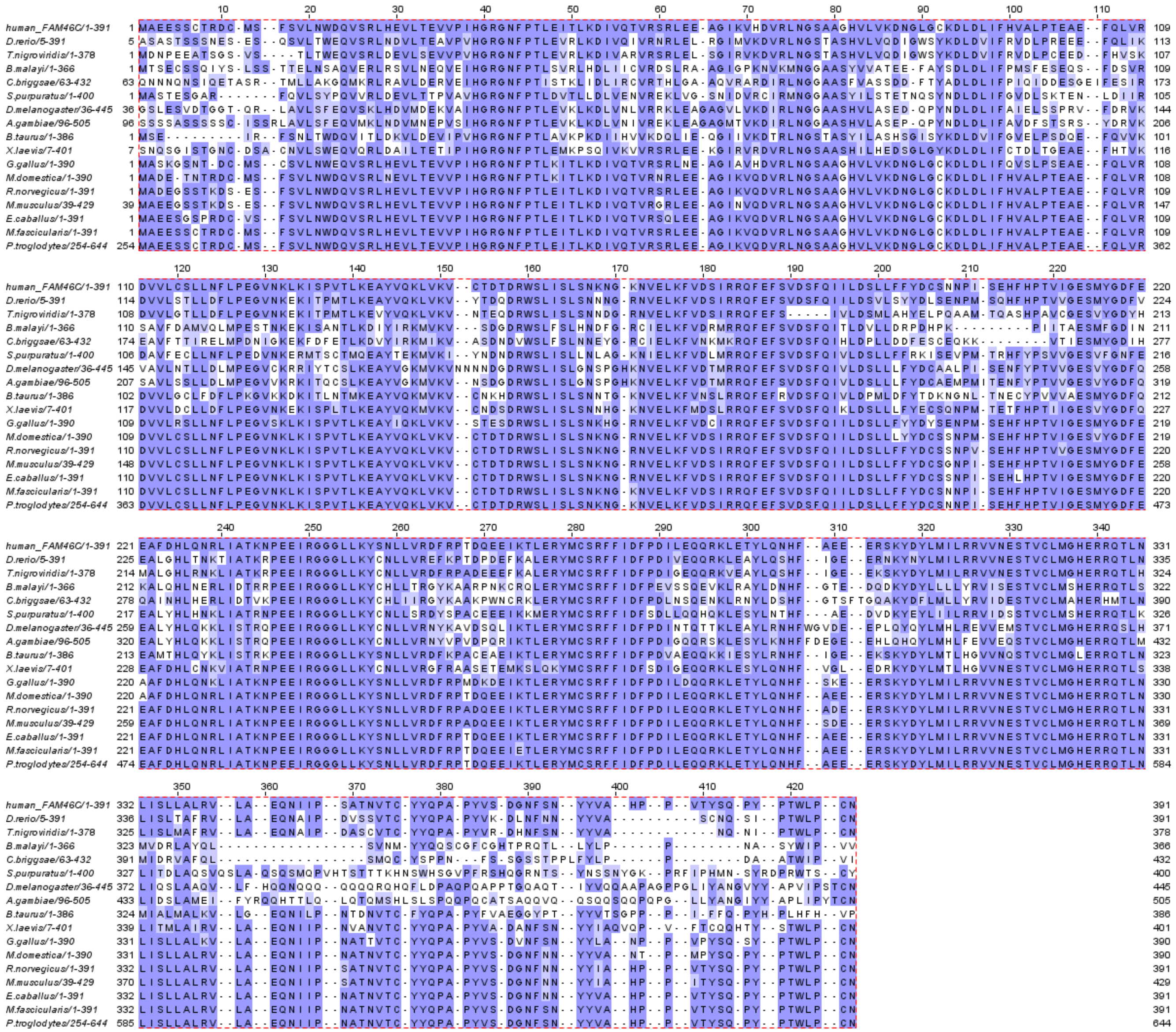

Supplementary Figure 1

C

	FAM46A	FAM46B	FAM46C	FAM46D
FAM46A	100.0	61.5	71.3	60.0
FAM46B	61.5	100.0	61.7	51.1
FAM46C	71.3	61.7	100.0	58.5
FAM46D	60.0	51.1	58.5	100.0

D

	Interactors in Y2H screens
FAM46A	ZC3H10, SNRPC, ROR2, GLIS2, DMRTB1, ARID5A, FAM46B, SERF2, RHOXF2, KRTAP26-1, TOLLIP, YPEL3, RNF44
FAM46B	SMAP2, MKRN3, BHLHE40, ENKD1, ZC3H10, KLHL38, HNRNPF, TBX6, DAZAP2, PSMF1, SOX5, ZNF503, OTX2, USP54, C10orf55, MGAT5B, FOSB, RBPMS2, AES, YPEL3, CERCAM, POU2AF1, **PLK4**, RUSC1, ZNF688, KRTAP6-2, UBAP2, ACY1, KRTAP19-6, SLC27A4, CYSRT1, KRT34, POU6F2, NICN1, TNS2, PSMB8, NAF1, AKAP9, FAM168B, FOS, RFX6, NR1D2, RIMBP3C, RHOXF2, FAM46A, SLC15A2, TRIP13, SOHLH1, HIVEP1, PROP1, FOXI1, SPAG8, UBQLN2, AKAP8L, ZBTB2, HMGXB4, LINC01600, H1FX-AS1, GUSBP5, AC099791.1, TP73-AS1, CLUHP3, SLC25A51P1, AC104452.1
FAM46C	DAZAP2, TRIP6, **PLK4**, AP2B1, RHOXF2
FAM46D	RHOXF2, C1orf94, DYDC2, PLEKHG4, KRTAP11-1, PTPN3, MSX2, RASSF3, PABPC3, ARID51, KRTAP6-2, HEY1, C10orf55

Supplementary Figure 1. FAM46C/TENT5C conservation across species and similarity between human FAM46/TENT5 family members. **a)** Phylogenetic tree of FAM46/TENT5 family protein sequences showing conservation of FAM46C/TENT5C across species. The family of FAM46 proteins was recently renamed TENT5 in recognition of *bona fide* nucleotidyl transferase activity. Scale bar represents relative phylogenetic distance. **b)** Multiple sequence alignment of FAM46C proteins using Clustal Omega (https://www.ebi.ac.uk/Tools/msa/clustalo/), showing conservation at the amino acid level across multiple eukaryotic species. Amino acid identity between *D. Melanogaster* and *H. Sapiens* is 57%, by Clustal Omega. **c)** Amino acid percent identity of human FAM46C paralogs, created by Clustal 2.1 Matrix (https://www.ebi.ac.uk/Tools/msa/clustalo/). For alignment and assignment of nucleotidyltransferase domain, please see[1]. **d)** Protein interactors of the four human FAM46/TENT5 family members, as identified in HuRI: The Human Reference Protein Interactome Mapping Project v3 (http://interactome.baderlab.org).

Supplementary Figure 2

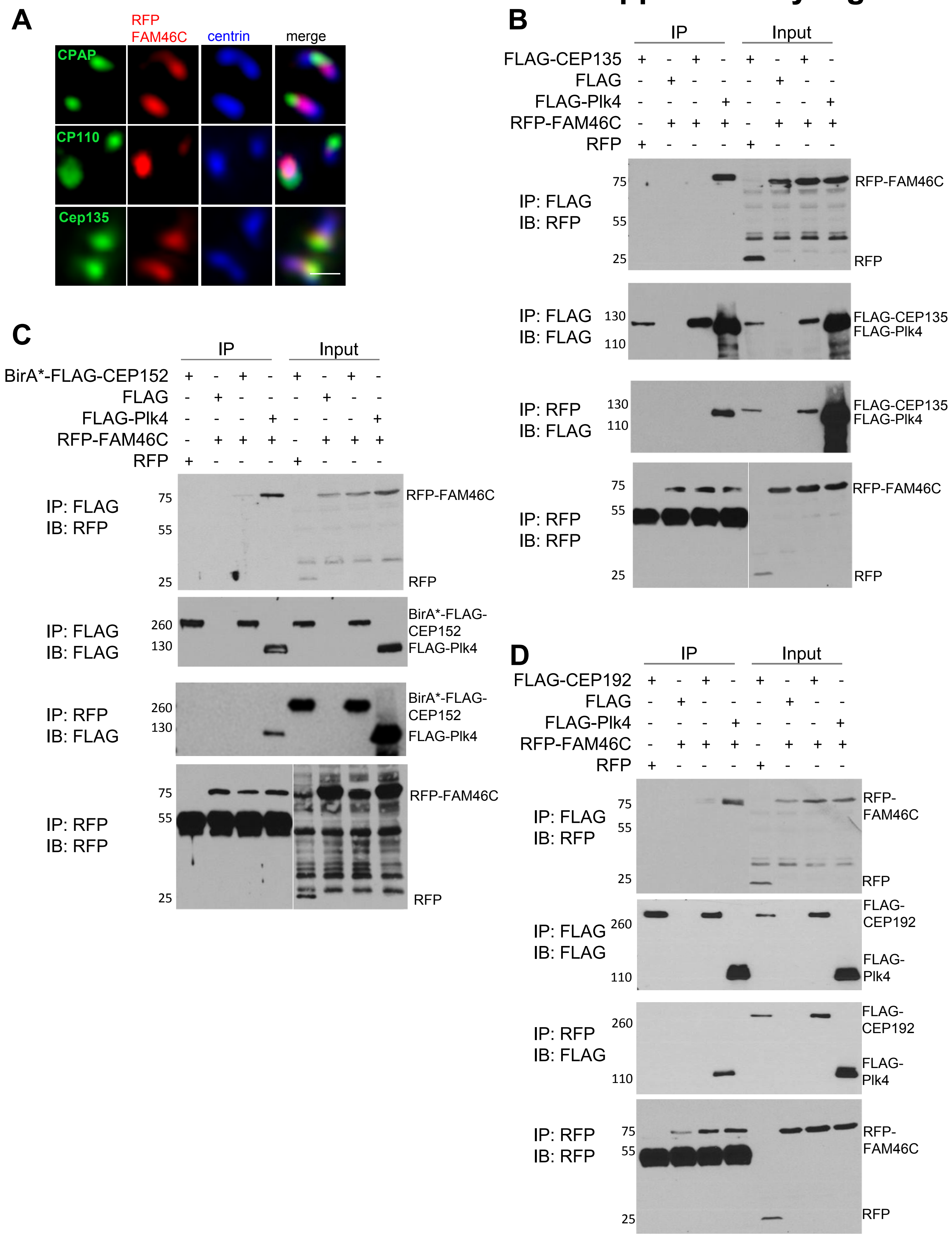

Supplementary Figure 2. FAM46C does not physically interact with centriolar scaffolding proteins CEP135, CEP152, and CEP192. a) Representative immunofluorescence images of U2OS cell centrosomes labeled with the indicated antibodies, showing relationship of centriolar protein staining to that of FAM46C. Bar 1µm. **b-d)** Immunoblots of RFP-FAM46C and the indicated proteins after coexpression in HEK293T cells, showing no evidence of physical interaction between RFP-FAM46C and FLAG-CEP135 **(b)**, BirA*- FLAG-CEP152 **(c),** or FLAG-CEP192 **(d).** The interaction between RFP-FAM46C and FLAG-Plk4 provided a positive control in these co-immunoprecipitation experiments **(b-d).**

Supplementary Figure 3

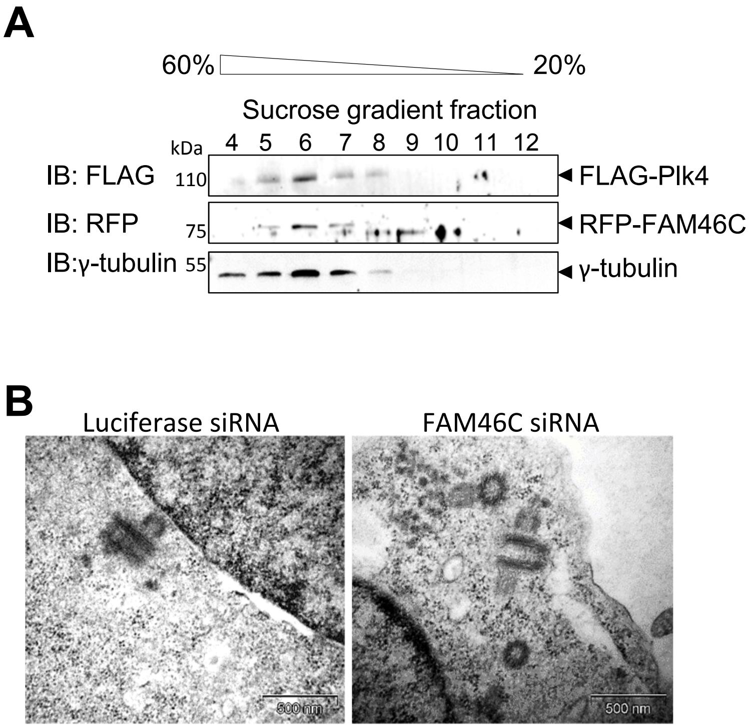

Supplementary Figure 3. FAM46C is a centrosomal protein that regulates centriole number. a) Representative immunoblot showing presence of FAM46C and Plk4 in centrosomal fractions isolated from proliferating U2OS cells. Cells that had been co-transfected with FLAG-Plk4 and RFP-FAM46C were harvested in exponential growth phase and cell lysates subjected to discontinuous sucrose gradient fractionation[2] (% sucrose as indicated). Proteins in each of 14 fractions were separated by SDS/PAGE, followed by immunoblot with FLAG, RFP and γ-tubulin (centrosomal fractions) antibodies. RFP-FAM46C and FLAG-Plk4 were concentrated in the same fractions (lanes labelled 5-7, sucrose ≈ 45-55%), in which γ-tubulin was also enriched. **b)** Transmission electron micrography reveals increased number of structurally intact centrioles with FAM46C knockdown using siRNA in HEK293T cells.

Supplementary Figure 4

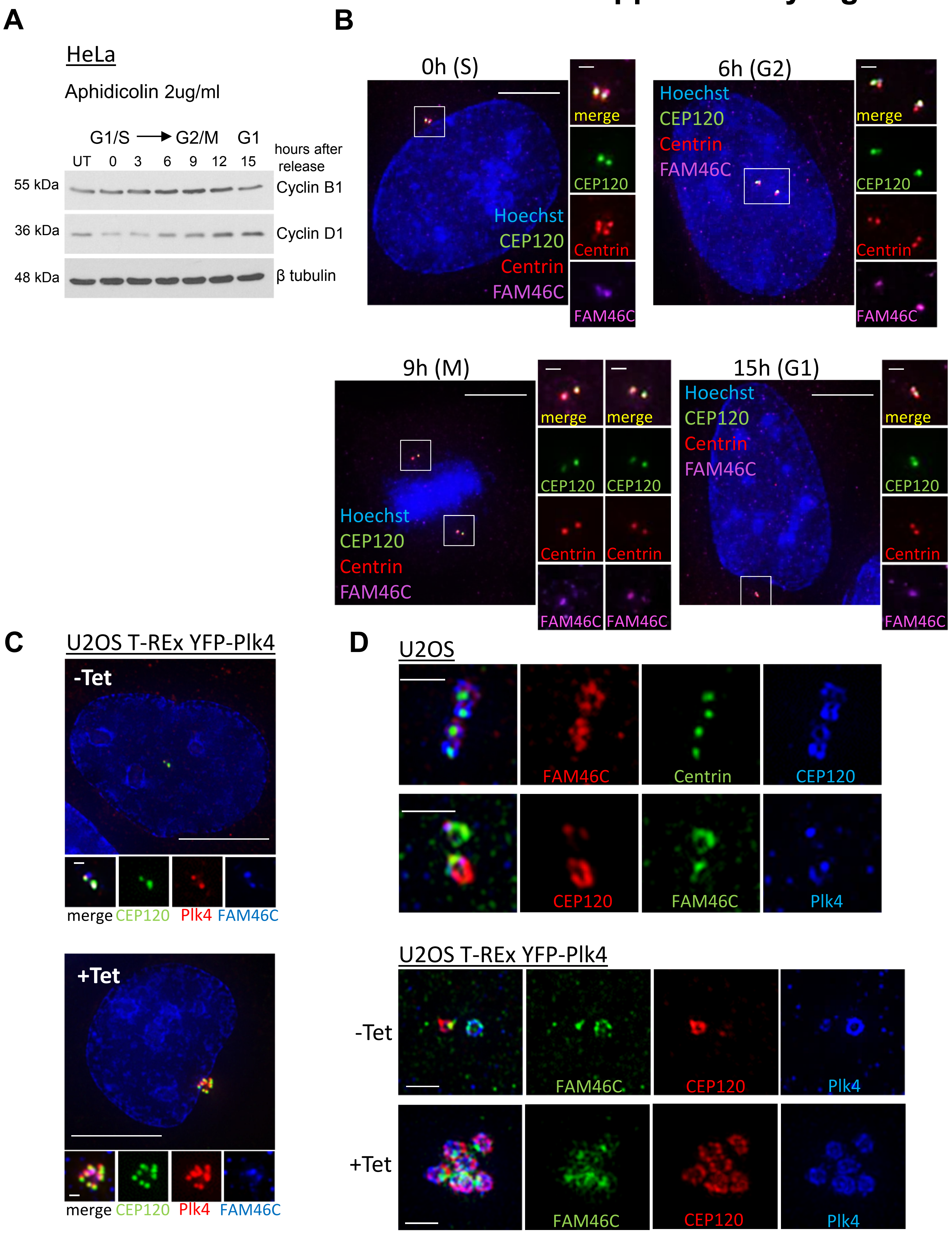

Supplementary Figure 4. FAM46C localizes predominantly to the mother centriole, throughout the cell cycle. a) Representative immunoblots of extracts from synchronized HeLa cells following release of cell cycle arrest after treatment with Aphidicolin X48h, using antibodies to the indicated cell cycle proteins. Phases of the cell cycle are deduced from temporal expression of cyclin B1 and cyclin D1, as shown. **b)** Representative immunofluorescence images of HeLa cells released from cell cycle arrest, as in **a)**, labeled with antibodies to: CEP120 (green), centrin (red) and FAM46C (blue), and with Hoechst. The right panels/inserts show magnified centrosomes (boxed in white). At the arrest in G1/S (time 0), two centrosomes consisting of mother-daughter paired centrioles (4 centrin positive foci) are visualized. CEP120 stains daughter centrioles preferentially, while FAM46C staining is observed predominantly at the other centriole (i.e. mother). As cells progress through S and into G2 (6h), the centrosomes separate and FAM46C staining persists at the mother centrioles. Localization of FAM46C predominantly to the mother centriole was also observed in M (9h) and G1 (15h). **c)** Distinct localization of FAM46C to the mother centrioles is seen in the Plk4-induced centriole overduplication system. Representative immunofluorescence images of U2OS T-REx YFP-Plk4 cells stained with antibodies to CEP120 (green), Plk4 (red) and FAM46C (blue), with Plk4 overexpression induced by tetracycline X24h (+Tet) in bottom panel. With YFP-Plk4 expression, there is a centriole overduplication phenotype with multiple daughter centrioles, as identified by CEP120 staining, surrounding two FAM46C positive foci (mother centrioles) in a rosette configuration. Plk4 staining was observed at all centrioles. Bars: 10μm, inset 1μm. **d)** Localization of FAM46C to mother centrioles is confirmed in U2OS cells imaged at super resolution. Merged and individual channel 3D-SIM micrographs of centrosomes in unsynchronized U2OS (top panels) or U2OS T-REx YFP-Plk4 cells (bottom panels, with and without addition of Tetracycline) labelled with antibodies against centrin (green), FAM46C (U20S: red (top) and green (bottom); U20S T-Rex: green), CEP120 (U2OS: blue

(top) and red (bottom); U20S T-Rex: red), and Plk4 (blue). Bars 1μm.

Supplementary Figure 5

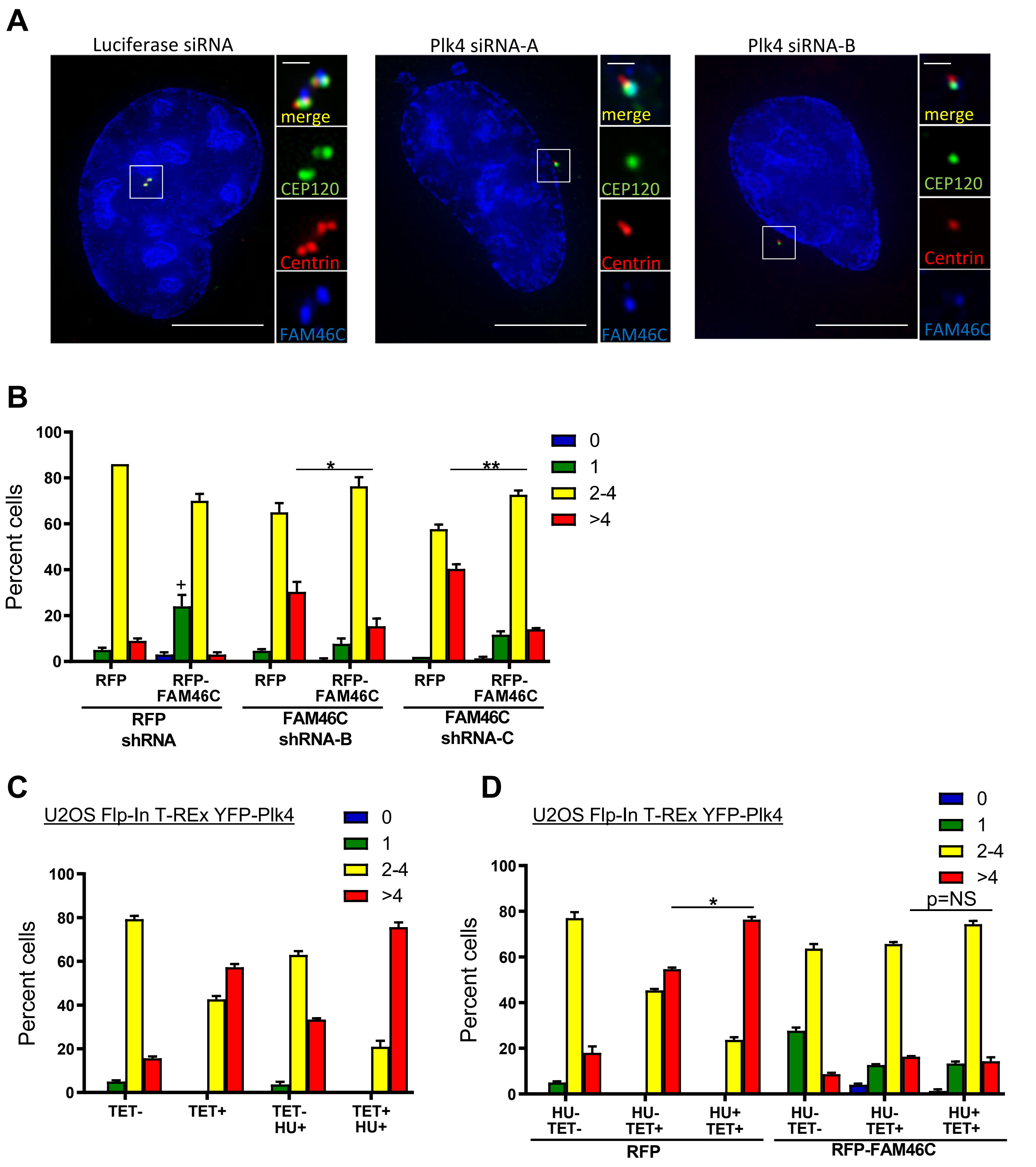

Supplementary Figure 5. FAM46C knockdown induces centriolar amplification. a) In U2OS cells depleted of Plk4 using siRNA for 48h, the majority of cells display single centrioles. Representative immunofluorescence images show that the residual centriole stains positive for FAM46C. Bars: 10µm, inset 1µm. **b)** Bar graph showing proportion of U2OS cells with indicated number of centrioles per cell, quantified by scoring centrin-positive foci, showing partial rescue of the centriole overduplication phenotype seen in FAM46C shRNA cells after transfection with RFP-FAM46C, n=3 independent experiments with >50 cells measured for each condition, $^{+}$p= 0.06, RFP shRNA: RFP-FAM46C vs. RFP; *p=0.055, FAM46C shRNA-B: RFP-FAM46C vs. RFP; **p=0.004: FAM46C shRNA-C: RFP-FAM46C vs. RFP. Data are means ±SEM. **c)** Augmentation of Plk4 overduplication phenotype by HU arrest of U2OS T-REx YFP-Plk4 cells with Plk4 expression induced by tetracycline (TET+). Bar graph shows proportion of cells with indicated number of centrioles per cell, quantified by scoring centrin-positive foci. n=3 independent experiments with 80 cells measured in each. **d)** Suppression of HU-augmented Plk4 overduplication phenotype by RFP-FAM46C in U2OS T-REx YFP-Plk4 cells with Plk4 expression induced by tetracycline (TET+) and transfected with RFP or RFP-FAM46C X40h. Bar graph shows proportion of cells with indicated number of centrioles per cell, quantified by scoring centrin-positive foci. n=3 independent experiments with 80 cells measured in each, *p<0.01 vs. RFP HU- TET+. P=NS vs. RFP-FAM46C HU-TET+. Data are means ±SEM.

Supplementary Figure 6

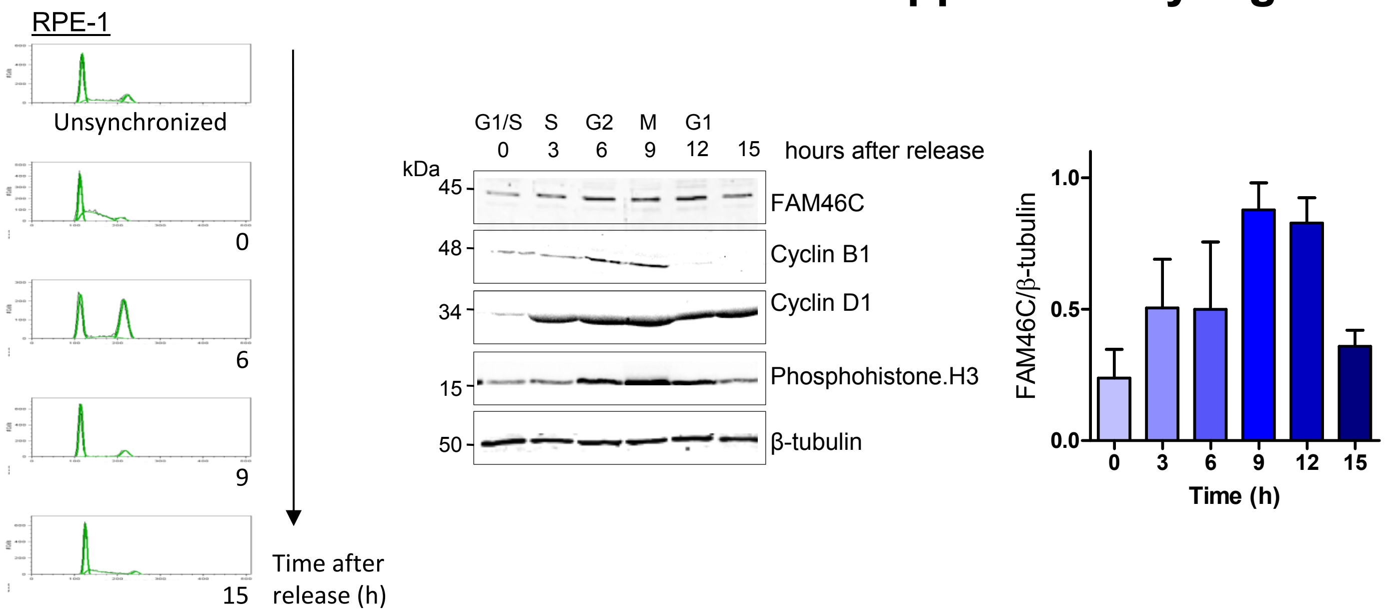

Supplementary Figure 6. Cell cycle dependent expression of endogenous FAM46C. Flow cytometry analysis of RPE-1 cells demonstrated timing of stages of the cell cycle following release from double thymidine arrest (left panel). Representative immunoblots of extracts from synchronized RPE-1 cells, using the indicated antibodies (middle panel). Phases of the cell cycle are deduced from flow cytometry traces combined with temporal expression of cell cycle proteins, as shown. Quantification of FAM46C protein expression at the indicated time after release, relative to β-tubulin control (right panel), shows that FAM46C level is cell cycle- dependent and peaks at ≈M phase (n=3).

Supplementary Figure 7

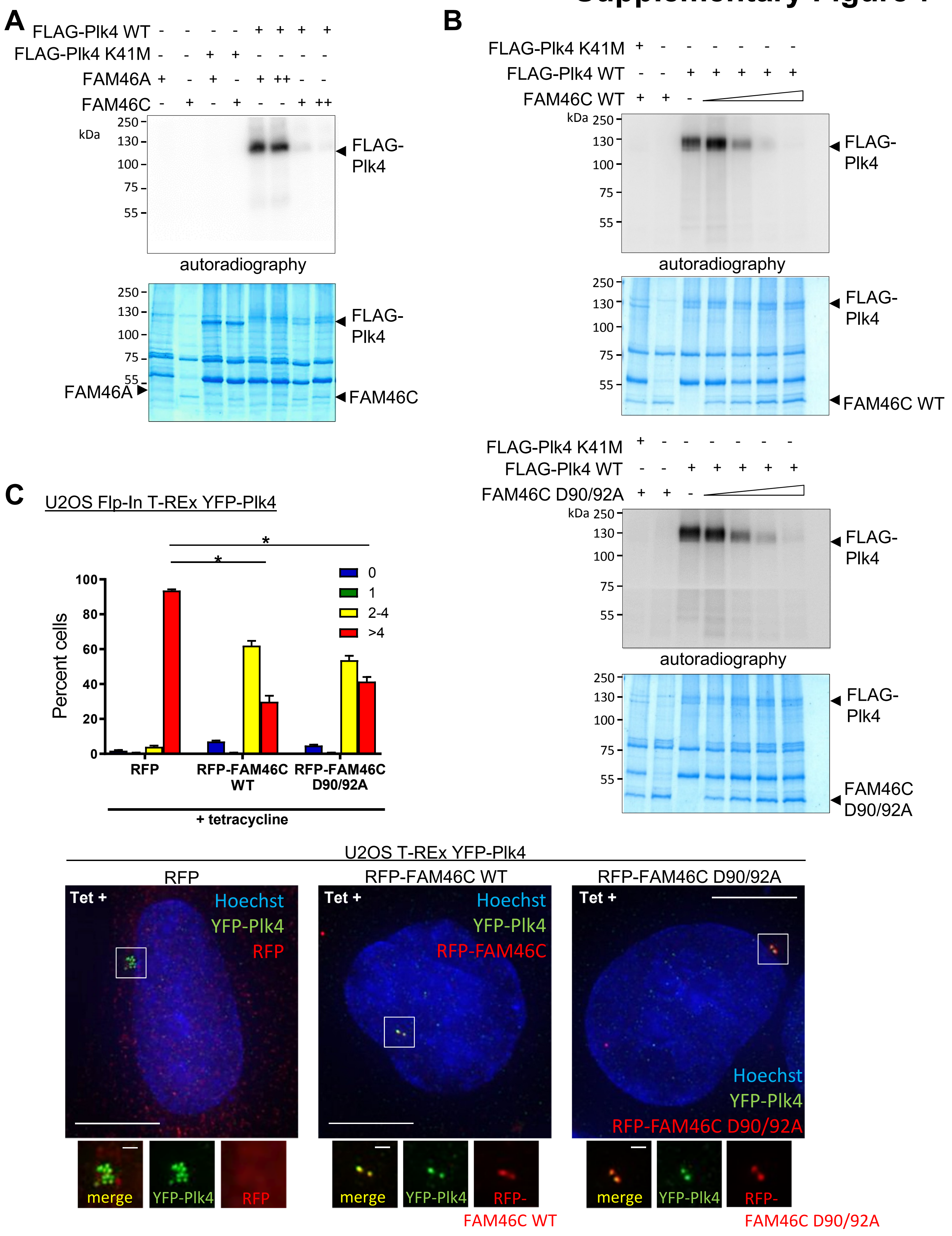

Supplementary Figure 7. Inhibition of Plk4 kinase activity and Plk4-driven centriole duplication by mutant FAM46C that lacks nucleotidyl transferase activity. a) *In vitro* kinase assay shows inhibition of wild-type (WT) FLAG-Plk4 autophosphorylation by FAM46C (wild-type), but not paralog FAM46A. Corresponding colloidal blue-stained gel shows input proteins. Autoradiographs show incorporation of γ-^{32}P during incubation, reflecting active phosphorylation. The kinase dead construct FLAG-Plk4 K41M lacks autophosphorylation. **b)** *In vitro* kinase assay showing dose-dependent reduction in wild-type (WT) Plk4 autophosphorylation by FAM46C WT (top panels) and also to a comparable extent by catalytically inactive FAM46C D90/92A, which lacks nucleotidyltransferase activity (bottom panels). Increasing amounts of FAM46C WT or FAM46C D90/92A are indicated by wedges, specified for each lane under the colloidal blue-stained gels of input proteins. Autoradiographs show incorporation of γ-^{32}P during incubation, reflecting active phosphorylation. **c)** Suppression of Plk4 centriole overduplication phenotype by FAM46C WT and also by catalytically inactive FAM46C D90/92A mutant, shown in representative immunofluorescence images of U2OS T-REx YFP-Plk4 cells with Plk4 (green) expression induced by tetracycline (Tet+) and transfected with RFP, RFP-FAM46C WT or RFP-FAM46C D90/92A (red) X42h (bottom panels). Cells were stained with Hoechst (blue), and RFP (red) and YFP (green) imaged directly (bottom panels). The inserts show magnified centrosomes (boxed in white) for each condition. Bar graph (top panel) shows proportion of cells with indicated number of centrioles per cell, quantified by scoring Plk4 positive foci. n=3 independent experiments with 80 cells measured in each, *$p<0.05$ vs. RFP alone.

Supplementary Figure 8

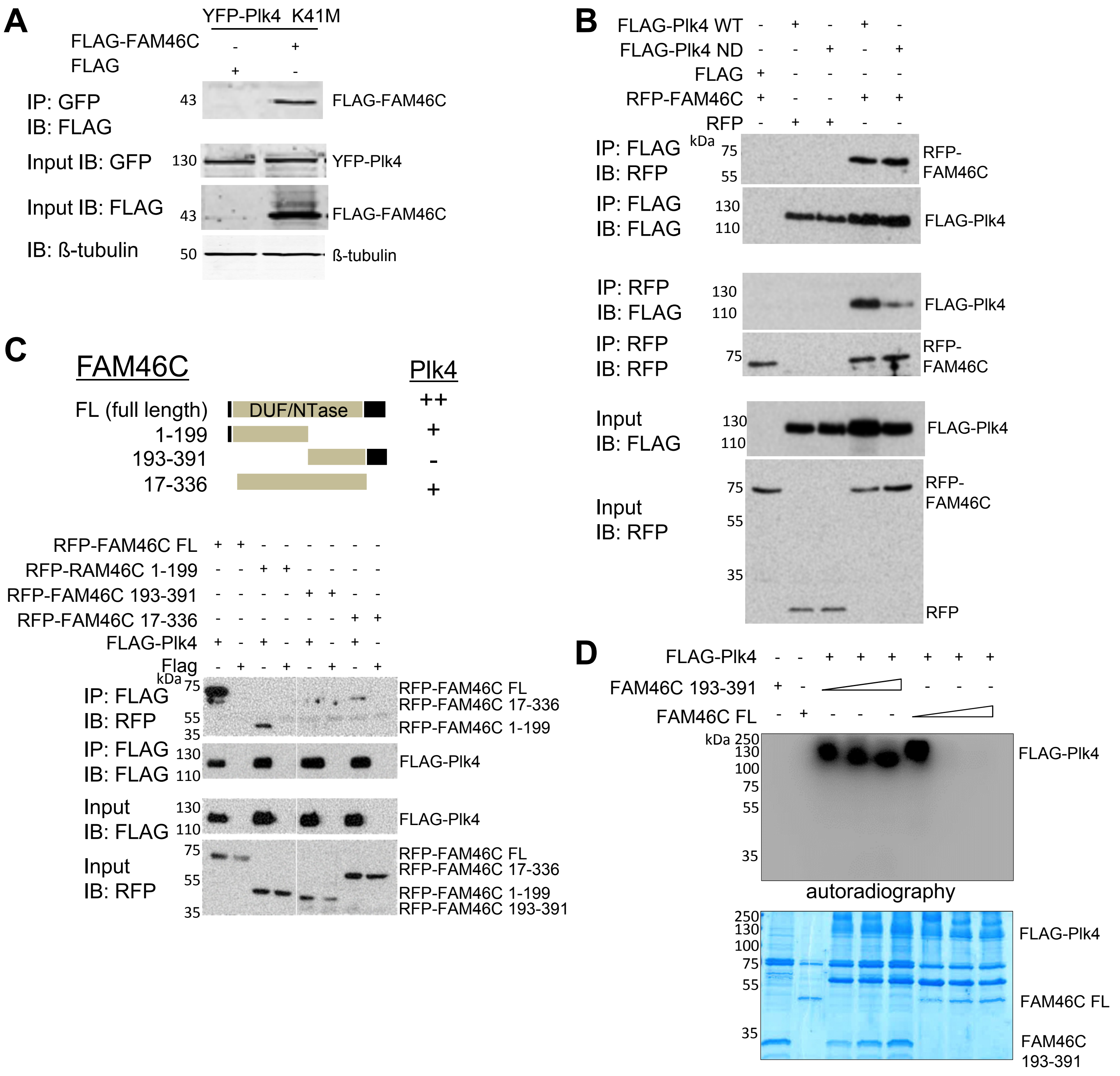

Supplementary Figure 8. Dependencies of FAM46C and Plk4 interaction, examined using deletion constructs and mutant Plk4s in co-immunoprecipitation experiments. a) Immunoblots of kinase dead YFP-Plk4 K41M and FLAG-FAM46C after coexpression in HEK293T cells, showing physical interaction between FLAG-FAM46C and kinase dead Plk4. **b)** Immunoblots of RFP-FAM46C with wild-type (WT) and nondegradable (ND) FLAG-Plk4 (Ser293A and Thr297A) after coexpression in HEK293T cells, showing physical interaction between RFP-FAM46C and nondegradable Plk4. **c)** Schematic diagram of full length FAM46C (FL) and FAM46C deletion constructs and summary of domain-dependent interactions with full length wild-type Plk4 (top panel), and immunoblots of full length wild-type FLAG-Plk4 and the indicated FAM46C fragments, after coexpression in HEK293T cells (bottom panel), showing interaction between FLAG-Plk4 and RFP-FAM46C N-terminal/NTase domain fragments1-199 and 17-336, but not RFP-FAM46C C-terminal fragment 193-391. **d)** *In vitro* kinase assay shows inhibition of wild-type Plk4 autophosphorylation by full length (FL) FAM46C but no effect of comparable amounts of the non-interactive FAM46C 193-391 C-terminal fragment. Increasing amounts of FAM46C 193-391 or full length FAM46C are indicated by wedge. Corresponding colloidal blue-stained gel shows input proteins. Autoradiographs show incorporation of γ-^{32}P during incubation, reflecting active phosphorylation.

Supplementary Figure 9

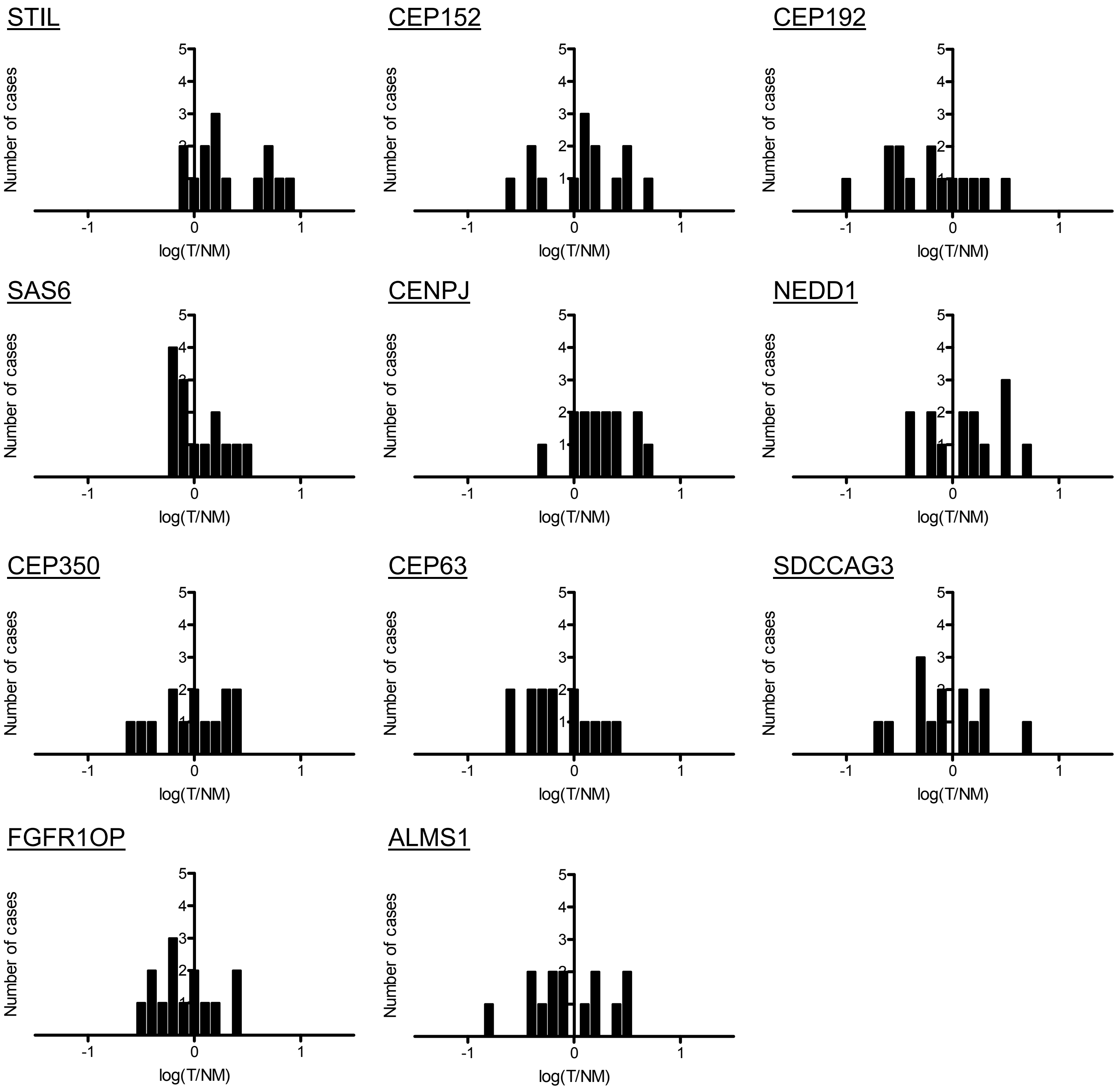

Supplementary Figure 9. Expression of Plk4 interactome centrosomal proteins in human colorectal cancer. Distribution of expression of centrosomal proteins known or predicted to interact with Plk4 by BioID[3] in colorectal primary tumour (T) compared with paired normal mucosa (NM) in microdissected specimens from 13 cases of high risk colorectal cancer. T/NM ratio is displayed on a log scale. Expression was determined by qPCR, relative to the control GAPDH. Each patient case is represented by a single vertical unit. There is no apparent depletion of any of these Plk4 interactors in tumour tissue, whereas FAM46C was consistently depleted in the same specimens (see Fig. 6a, bottom panel). T/NM ratio is displayed on a log scale.

Supplementary Figure 10

Figure 1b

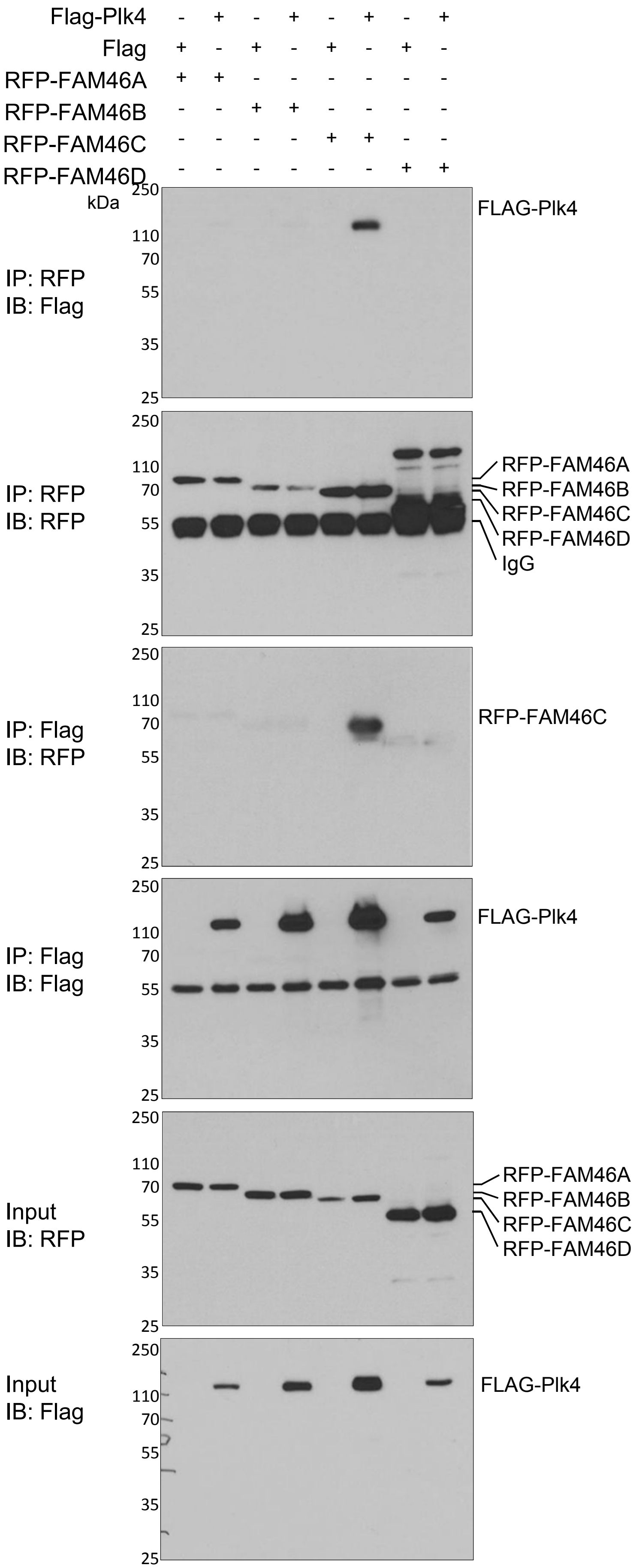

Figure 1d

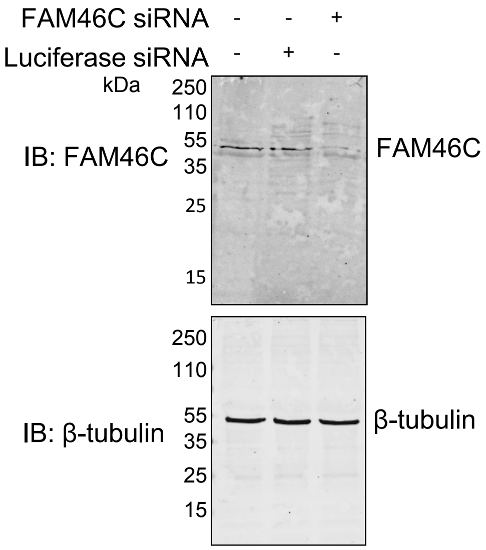

Figure 1e

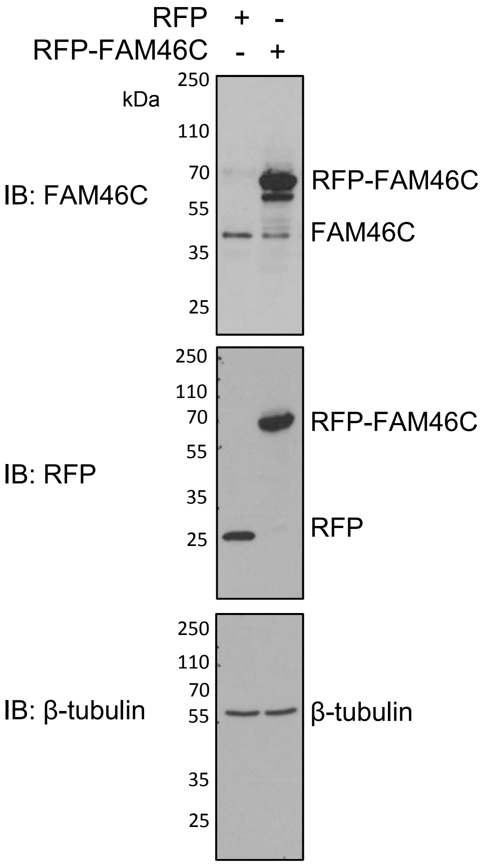

Supplementary Figure 10

Figure 2a

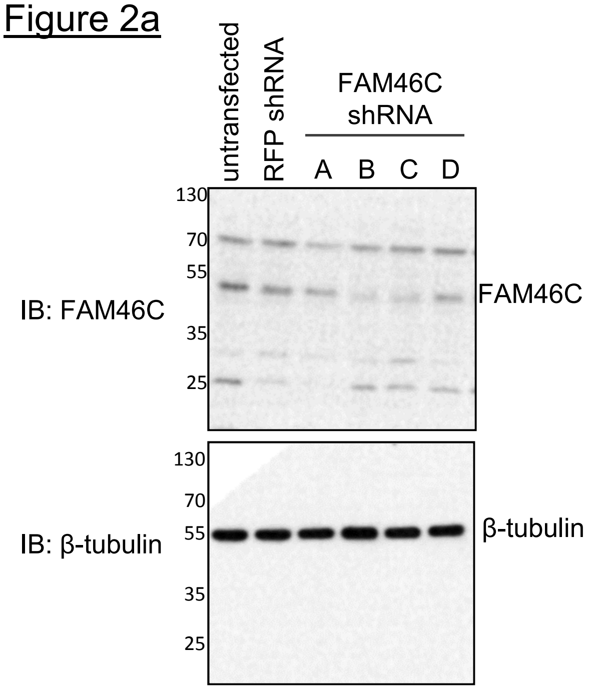

Figure 4c

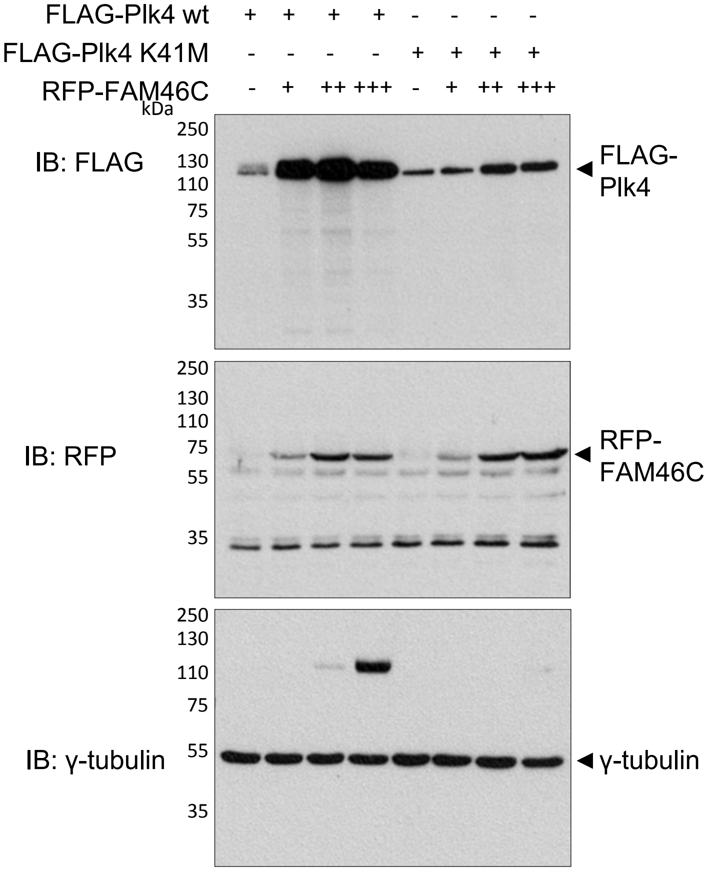

Supplementary Fig. 3a

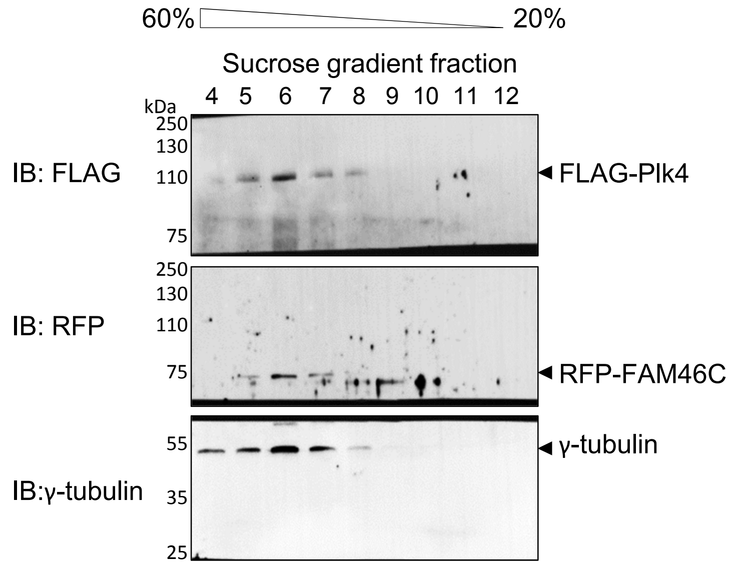

Supplementary Fig. 4a

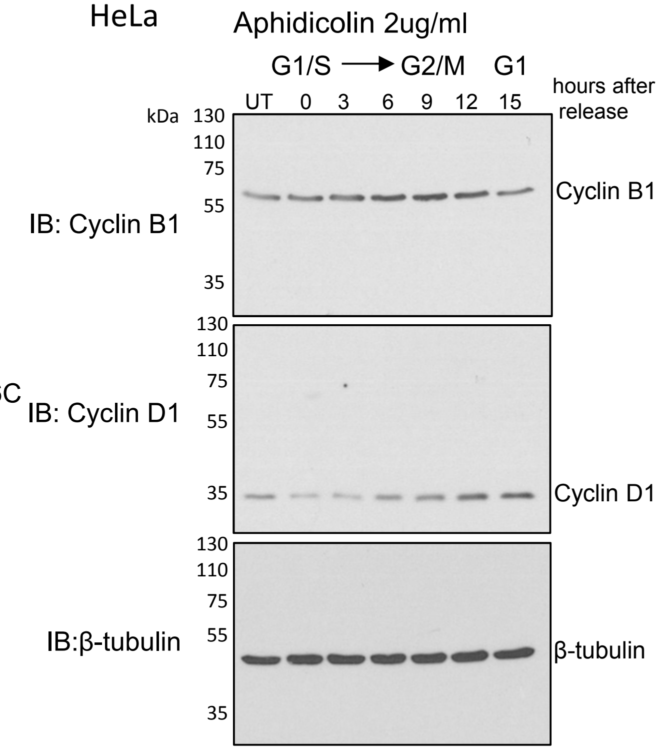

Supplementary Figure 10

Supplementary Fig. 8a

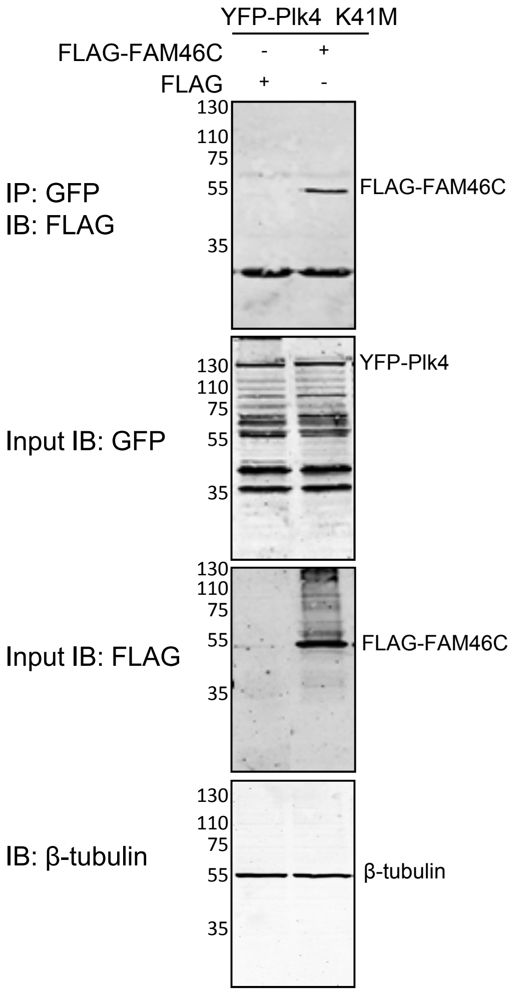

Supplementary Fig. 8b

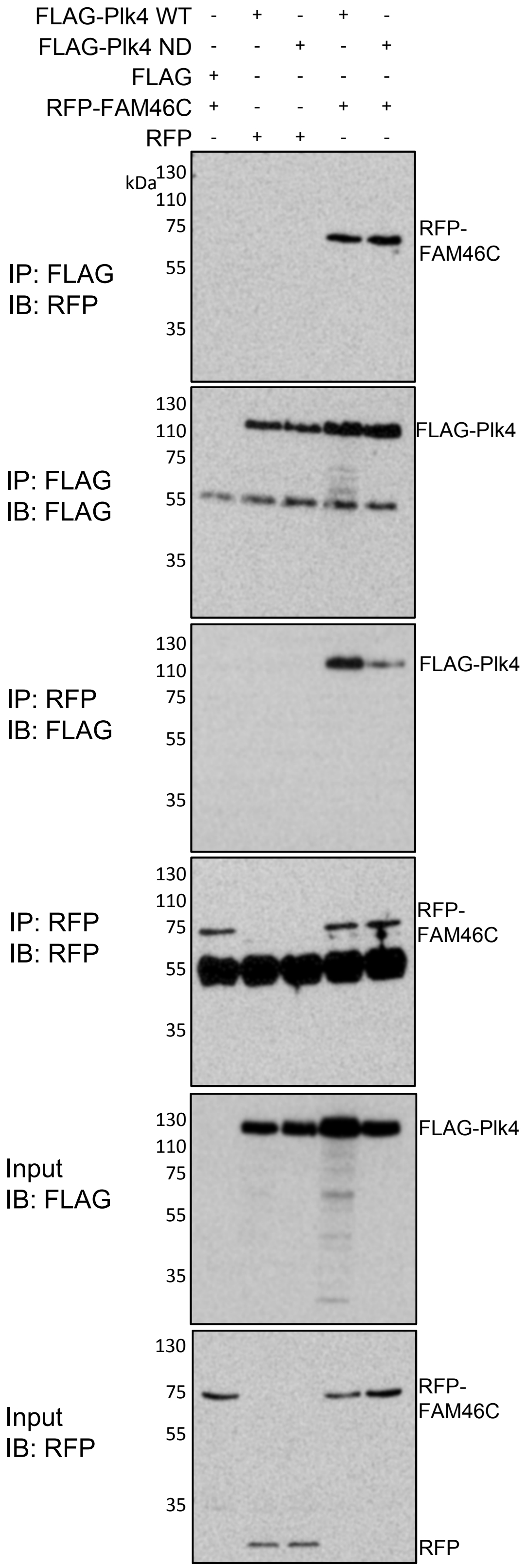

Supplementary Figure 10. Full uncropped Immunoblot images from main and supplementary figures.

Supplementary Tables

Supplementary Table 1

Supplemental Table 1. Clinicopathologic characteristics of 13 colorectal cancer specimens

Variable	Data
Age, median (range)	53.8 (35-78)
Sex (M:F)	8:5
Location	
Colon, right	2 (15%)
Colon, left/sigmoid	6 (46%)
Rectum	5 (39%)
AJCC Stage*	
I	1 (8%)
II	2 (15%)
III	2 (15%)
IV	8 (62%)
T*	
1	0 (0%)
2	2 (15%)
3	9 (69%)
4	2 (15%)
N*	
0	4 (31%)
1	3 (23%)
2	6 (46%)
Differentiation	
Poor	4 (31%)
Well/Moderately well	9 (69%)

*AJCC, American Joint Committee on Cancer (2006)

Supplementary Table 1. Patient demographics and clinicopathologic features for 13 colorectal cancer specimens. The banked primary tumour and normal mucosa specimens analyzed in Fig. 6a,b,c and f were derived from 13 patients who either had synchronous liver metastases at presentation (n=8) or who developed liver metastases subsequently (n=5).

Supplementary Table 2

PLK4 related genes
ARHGEF1
ECT2
CEP85
FAM46C
PREX2
PLK1
PLK2
PLK3
PLK4
PLK5

Supplementary Table 2. Plk4 related genes not present in the BioID Plk4 interactome[3], assayed using qPCR in 13 colorectal cancer specimens described in Table S1.

Supplementary References

1 Kuchta, K. *et al.* FAM46 proteins are novel eukaryotic non-canonical poly(A) polymerases. *Nucleic acids research* **44**, 3534-3548, doi:10.1093/nar/gkw222 (2016).

2 Gogendeau, D., Guichard, P. & Tassin, A. M. Purification of centrosomes from mammalian cell lines. *Methods in cell biology* **129**, 171-189, doi:10.1016/bs.mcb.2015.03.004 (2015).

3 Kazazian, K. *et al.* Plk4 Promotes Cancer Invasion and Metastasis through Arp2/3 Complex Regulation of the Actin Cytoskeleton. *Cancer research* **77**, 434-447, doi:10.1158/0008-5472.CAN-16-2060 (2017).
