# Supplementary material for: FAM46C/TENT5C functions as a tumor suppressor through inhibition of Plk4 activity
Source: Commun Biol. 2020 Aug 17;3:448. doi: 10.1038/s42003-020-01161-3 (PMC7431843; doi:10.1038/s42003-020-01161-3)
Supplement: Supplementary file 2 — Description of Additional Supplementary Files [file 42003_2020_1161_MOESM2_ESM.pdf]

Description of Additional Supplementary Files

File Name: **Supplementary Data 1**
Description: the source data for the Figures and Tables
