# Supplementary material for: FAM46C/TENT5C functions as a tumor suppressor through inhibition of Plk4 activity
Source: Commun Biol. 2020 Aug 17;3:448. doi: 10.1038/s42003-020-01161-3 (PMC7431843; doi:10.1038/s42003-020-01161-3)
Supplement: Supplementary file 4 — Reporting Summary [file 42003_2020_1161_MOESM4_ESM.pdf]

nature research

Corresponding author(s): Carol Swallow
COMMSBIO-19-0751C

Last updated by author(s): 2020/06/26

Reporting Summary

Nature Research wishes to improve the reproducibility of the work that we publish. This form provides structure for consistency and transparency in reporting. For further information on Nature Research policies, see Authors & Referees and the Editorial Policy Checklist.

Statistics

For all statistical analyses, confirm that the following items are present in the figure legend, table legend, main text, or Methods section.

n/a	Confirmed	
☐	☒	The exact sample size (n) for each experimental group/condition, given as a discrete number and unit of measurement
☐	☒	A statement on whether measurements were taken from distinct samples or whether the same sample was measured repeatedly
☐	☒	The statistical test(s) used AND whether they are one- or two-sided<br>*Only common tests should be described solely by name; describe more complex techniques in the Methods section.*
☒	☐	A description of all covariates tested
☐	☒	A description of any assumptions or corrections, such as tests of normality and adjustment for multiple comparisons
☐	☒	A full description of the statistical parameters including central tendency (e.g. means) or other basic estimates (e.g. regression coefficient) AND variation (e.g. standard deviation) or associated estimates of uncertainty (e.g. confidence intervals)
☐	☒	For null hypothesis testing, the test statistic (e.g. F, t, r) with confidence intervals, effect sizes, degrees of freedom and P value noted<br>*Give P values as exact values whenever suitable.*
☒	☐	For Bayesian analysis, information on the choice of priors and Markov chain Monte Carlo settings
☒	☐	For hierarchical and complex designs, identification of the appropriate level for tests and full reporting of outcomes
☒	☐	Estimates of effect sizes (e.g. Cohen's d, Pearson's r), indicating how they were calculated

Our web collection on statistics for biologists contains articles on many of the points above.

Software and code

Policy information about availability of computer code

Data collection	Based on multiple sequence alignments generated by Clustal Omega (https://www.ebi.ac.uk/Tools/msa/clustalo/), an unrooted phylogenetic tree was generated for select FAM46C orthologs and the human FAM46C paralogs, FAM46 A, B and D (2019). Transcriptome and clinical datasets from TCGA-COAD and Kim et al. (Molecular Oncology, 2014) were downloaded using the NIH National Cancer Institute GDC Data Portal Data release 8.0 https://portal.gdc.cancer.gov/, cBioPortal for Cancer Genomics v1.8.3 http://www.cbioportal.org/ and CRN Nexus http://syslab4.nchu.edu.tw/.
Data analysis	Prism software (GraphPad Software, La Jolla, CA)

For manuscripts utilizing custom algorithms or software that are central to the research but not yet described in published literature, software must be made available to editors/reviewers. We strongly encourage code deposition in a community repository (e.g. GitHub). See the Nature Research guidelines for submitting code & software for further information.

Data

Policy information about availability of data

All manuscripts must include a data availability statement. This statement should provide the following information, where applicable:
- Accession codes, unique identifiers, or web links for publicly available datasets
- A list of figures that have associated raw data
- A description of any restrictions on data availability

Data is presented in the manuscript of the text or web links are provided for publicly available datasets. No restrictions on data presented in this study.

Field-specific reporting

Please select the one below that is the best fit for your research. If you are not sure, read the appropriate sections before making your selection.

☒ Life sciences ☐ Behavioural & social sciences ☐ Ecological, evolutionary & environmental sciences

For a reference copy of the document with all sections, see nature.com/documents/nr-reporting-summary-flat.pdf

Life sciences study design

All studies must disclose on these points even when the disclosure is negative.

Sample size	No sample size calculation was performed.
Data exclusions	No data was excluded from the analyses.
Replication	All attempts at replication were successful in the provided data.
Randomization	Randomization was not relevant to the study.
Blinding	Blinding was done to the treatment of the cell lines for the tumour measurement in the mouse studies. The technician performing the measurements was blinded to the cell line used.

Reporting for specific materials, systems and methods

We require information from authors about some types of materials, experimental systems and methods used in many studies. Here, indicate whether each material, system or method listed is relevant to your study. If you are not sure if a list item applies to your research, read the appropriate section before selecting a response.

Materials & experimental systems

n/a	Involved in the study
☐	☒ Antibodies
☐	☒ Eukaryotic cell lines
☒	☐ Palaeontology
☐	☒ Animals and other organisms
☒	☐ Human research participants
☐	☒ Clinical data

Methods

n/a	Involved in the study
☒	☐ ChIP-seq
☒	☐ Flow cytometry
☒	☐ MRI-based neuroimaging

Antibodies

Antibodies used	Full length FAM46C was cloned using the pET system into a bacterial expression vector containing a C-term 6×His tag. Recombinant protein was purified from Escherichia coli using Ni-NTA beads (Novagen) and used for immunization; rabbit immune sera were affinity-purified using standard procedures (Pacific Immunology Corp.). Antibodies used for immunofluorescence in this study were FAM46C (generated for this study, as above, 1:200), centrin (clone 20H5, Millipore), pericentrin (Sigma), Plk4 (NB100-894, Novus Biologicals Canada and MABC544, Millipore), SAS-6 (sc-82360, Santa Cruz), CP110 (A301-343A-1, Bethyl Laboratories), CPAP and CEP135 (L. Pelletier laboratory, Lunenfeld Tanenbaum Research Institute, Toronto), CEP120 (M. Mahjoub laboratory at University of Washington in St. Louis), ODF2 (H00004957-M01, Cedarlane/Abnova). Secondary antibodies were conjugated to Alexa Fluor 488, 546, 594, 633, or 647 (Life Technologies). DNA was detected using Hoechst. YFP and mCherry/RFP were visualized directly. The antibodies used for immunoblotting were: anti-β-tubulin (Sigma-Aldrich), anti-γ-tubulin (Sigma-Aldrich), anti-FLAG M2 (F1804, Sigma-Aldrich, anti-RFP (Abcam), anti-mCherry (Abcam), anti-FAM46C (this study, 1:1000), anti-GFP (ab290, Abcam), anti-Cyclin B1 (4135, Cell Signaling), anti-Cyclin D1 (2926, Cell Signaling), anti-Phosphohistone H3 (9701, Cell Signaling).
Validation	Prior incubation of anti-FAM46C with antigen eliminated centriolar staining, and staining was markedly reduced by depletion of FAM46C using siRNA. The remainder of the antibodies used were commercially validated, and centriolar antibodies including Sas-6, CPAP, CP110, Plk4, centrin were validated in the L. Pelletier lab and provided for use in these experiments.

Eukaryotic cell lines

Policy information about cell lines

Cell line source(s)	HEK293T and MDA MB-435 cell lines were a kind gift from the Tony Pawson laboratory (Lunenfeld Tanenbaum Research Institute, Toronto), and U2OS cells were a kind gift from the Laurence Pelletier laboratory (Lunenfeld Tanenbaum Research Institute).

Authentication	Cell lines were not authenticated by our laboratory.
Mycoplasma contamination	Mycoplasma testing was negative.
Commonly misidentified lines (See ICLAC register)	MDA-MB 435, misnomer as a breast cancer cell line but more in keeping with melanoma. This does not affect the design or conclusions from their use.

Animals and other organisms

Policy information about studies involving animals; ARRIVE guidelines recommended for reporting animal research

Laboratory animals	5 week-old female NCr Nude mice (Taconic Biosciences).
Wild animals	Study did not involved wild animals.
Field-collected samples	Study did not involve samples collected from the field.
Ethics oversight	All protocols were approved by the Toronto Centre for Phenogenomics (TCP) Animal Care Committee.

Note that full information on the approval of the study protocol must also be provided in the manuscript.

Clinical data

Policy information about clinical studies

All manuscripts should comply with the ICMJE guidelines for publication of clinical research and a completed CONSORT checklist must be included with all submissions.

Clinical trial registration	N/A
Study protocol	*Note where the full trial protocol can be accessed OR if not available, explain why.*
Data collection	*Describe the settings and locales of data collection, noting the time periods of recruitment and data collection.*
Outcomes	*Describe how you pre-defined primary and secondary outcome measures and how you assessed these measures.*
